# Screening of Natural Molecules as Adjuvants to Topical Antibiotics to Treat *Staphylococcus aureus* from Diabetic Foot Ulcer Infections

**DOI:** 10.3390/antibiotics11050620

**Published:** 2022-05-04

**Authors:** Diana Oliveira, Anabela Borges, Maria J. Saavedra, Fernanda Borges, Manuel Simões

**Affiliations:** 1 LEPABE—Laboratory for Process Engineering, Environment, Biotechnology and Energy, Faculty of Engineering, University of Porto, 4200-465 Porto, Portugal; up201406841@fe.up.pt (D.O.); apborges@fe.up.pt (A.B.); 2 ALiCE—Associate Laboratory in Chemical Engineering, Faculty of Engineering, University of Porto, 4200-465 Porto, Portugal; 3 CIQUP—Department of Chemistry and Biochemistry, Faculty of Sciences, University of Porto, 4169-007 Porto, Portugal; fborges@fc.up.pt; 4 DEQ—Department of Chemical Engineering, Faculty of Engineering, University of Porto, 4200-465 Porto, Portugal; 5 CITAB—Centre for the Research and Technology for Agro-Environment and Biological Sciences, University of Trás-os-Montes e Alto Douro, 5001-801 Vila Real, Portugal; saavedra@utad.pt; 6 Department of Veterinary Sciences, School of Agrarian and Veterinary Sciences, University of Trás-os-Montes e Alto Douro, 5001-801 Vila Real, Portugal

**Keywords:** antibiotic adjuvants, combination, diabetic foot ulcers, phytochemicals and derivatives, *Staphylococcus aureus*, topical antibiotics

## Abstract

Diabetic foot ulcers (DFUs) are a common result of a complex secondary complication of diabetes *mellitus*. More than half of DFUs become infected due to frequent colonization with *Staphylococcus aureus*. The use of topical antibiotics is proposed, especially in combination with natural adjuvants, to minimize the negative impacts caused by generalized use of systemic antibiotics. In this study, 13 different phytochemicals—namely chalcone, juglone, cinnamic acid, trigonelline, Furvina—and four nitrovinylfuran derivatives—guaiazulene, α-bisabolol, farnesol and nerolidol—were selected to be tested as antibiotic enhancers. After minimum inhibitory and bactericidal concentration (MIC and MBC) determination of each molecule against different strains of *S. aureus*, including clinical isolates from diabetic foot wounds (CECT 976, Xu212, SA 1199B, RN4220, MJMC102, MJMC109, MJMC110 and MJMC111), their potentiation effects on the antibiotics fusidic acid, mupirocin, gentamicin, oxacillin and methicillin were evaluated through the disc diffusion method. Farnesol at sub-MIC was able to restore the activity of methicillin and oxacillin on the MJMC102 and MJMC111 strains, as well as two MRSA clinical isolates, and potentiated the effect of the remaining antibiotics. The results obtained demonstrate the great potential for the topical application of phytochemicals and derivatives as antibiotic resistance modifier agents to combat multidrug resistance in bacterial wound infections.

## 1. Introduction

Diabetic foot ulcers (DFUs) are a skin breakage event caused by repetitive stress over an area, with the contribution of diabetic peripheral neuropathy in their development [1]. Peripheral neuropathy is a condition that causes nerve damage, triggered by the hyperglycemic state characteristic of diabetes [2]. This innervation damage may result in anatomical deformities that may cause skin fissures and therefore ulceration. DFUs are estimated to affect as many as 30% of diabetic patients during their lifetime [3]. Once DFUs are developed, there is a greater risk of amputation, with approximately 85% of all lower limb amputations (LLAs) in diabetes being preceded by foot ulcers [4].

The standard treatment applied to DFUs includes wound debridement, offloading (removing the load from the foot) and infection control [1]. Because half of DFUs become infected, control of the infection with antibiotics is crucial for wound healing success [5]. Routinely, this treatment comprises the use of systemic antibiotics that, apart from the necessity of higher doses to control the infection, are subject to limitations related to the concentration present at the wound site, as diabetic patients have limited lower extremity irrigation. Additionally, up to 53% of DFUs are colonized by multidrug-resistant microorganisms, especially biofilm-producing bacteria that are resistant to conventional antibiotic therapy [6]. The most frequently isolated microbes are *Staphylococcus aureus* and *Pseudomonas aeruginosa* [7,8]. These micro-organisms can exist either in planktonic or sessile states. Their ability to form biofilm is one of the modes of action by which bacteria exert antimicrobial tolerance [9]. Antibiotic-resistant microorganisms, with the lack of development of new drugs to overcome bacterial resistance mechanisms, represent one of the most severe threats to human health [10].

Nature has been a source of medicinal products since ancient times [11]. Plants yield a diverse group of secondary metabolites, such as phenolic compounds, essential oils, alkaloids, polypeptides and glycosides, each with a specific role in defense of the plant and each responsible for various therapeutic effects [12]. However, since the advent of antibiotics, the use of plant-based antimicrobials has been virtually non-existent, thus representing a largely unexploited resource. Despite some preliminary screening of new natural compounds from plant secondary metabolism (phytochemicals) to restock antibiotics, there was a quick withdrawal of this approach by the industry. Indeed, phytochemicals present a weaker antimicrobial activity compared to the available antibiotics. However, phytochemicals offer a richness of structural diversity and different modes of action, opening new horizons to face the paradigm of antibiotic resistance [10]. Additionally, due to synergistic interactions between phytochemicals and antibiotics, there is a potential to retrieve fewer effective antibiotics and deal with resistance phenomena. Therefore, following a significant innovation gap with regard to the discovery of new antimicrobials due to the application of ineffective discovery strategies and a deprioritization of antibacterial programs by the pharmaceutical industry, there is now a renewed interest in the application of plant natural products.

In the present study, we focus on the screening of different plant-based compounds as adjuvants to topical antibiotics to be used in the treatment of *S. aureus* DFU infections. To do so, 13 phytochemicals and derivatives (Table 1) belonging to different chemical classes, namely phenolics (chalcone, juglone and cinnamic acid), alkaloids (trigonelline), a synthetic nitrovinylfuran (furvina), as well as four 2-nitrovinylfuran derivatives and sesquiterpenoid constituents of essential oils (guaiazulene, α-bisabolol, farnesol and nerolidol) were selected. Their antibacterial activity per se, as well as their combined effect with the topical antibiotics fusidic acid, mupirocin, gentamicin, methicillin and oxacillin, were evaluated against different strains of *S. aureus*, including clinical isolates of MRSA and MSSA from diabetic foot wounds (CECT 976, Xu212, SA 1199B, RN4220, MJMC102, MJMC109, MJMC110 and MJMC111). Their antibacterial activity was evaluated by determination of the minimum inhibitory and bactericidal concentrations (MIC and MBC, respectively), and the combined effects of phytochemical-based molecules and antibiotics against *S. aureus* strains were assessed by the disc diffusion method. 

## 2. Results

In this study, 13 phytochemicals and their derivatives were first evaluated for their inhibitory (MIC) and bactericidal (MBC) activities against different strains of *S. aureus*, including clinical isolates of MRSA and MSSA from diabetic foot wounds (CECT 976, Xu212, SA 1199B, RN4220, MJMC102, MJMC109, MJMC110 and MJMC111) (Table 2). As shown in Table 2, juglone, furvina and farnesol were the molecules with the highest antibacterial activity, presenting the lowest MIC and MBC values. On the other hand, trigonelline, compound **2** (a nitrovinylfuran derivative) and cinnamic acid had no MIC or MBC found within the concentrations tested. In a second step, the effect of all the selected molecules on the activity of the antibiotics fusidic acid, mupirocin, gentamicin, oxacillin and methicillin was assessed through the disc diffusion method. The molecule was incorporated in an agar plate at a concentration 10 times lower than the MIC of each strain (in cases where MIC was detected) or at the maximum concentration tested (1000 mg/L). The mass of antibiotic incorporated in the disc was the same for all the strains studied and based on CLSI guidelines. 

**Figure 1:**
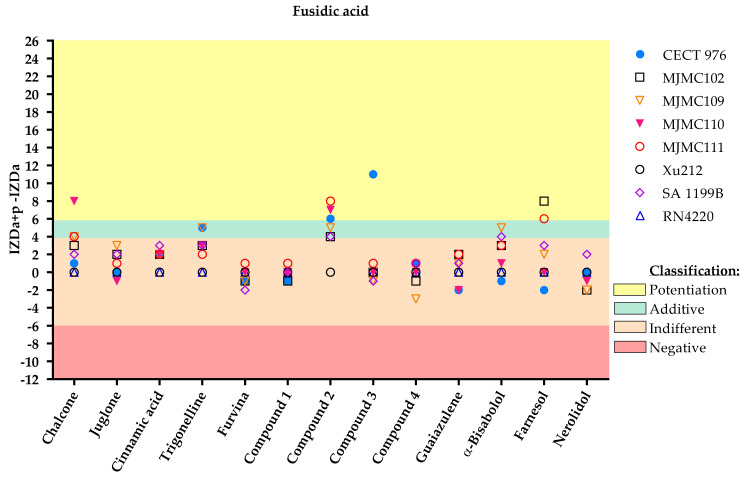
Inhibition zone diameter (IZD) values (mm) and respective classification according to the combined application of the selected phytochemicals and fusidic acid against the eight different *S. aureus* strains. The effect of dual combinations of antibiotics and phytochemicals was classified as potentiation when IZDa+p–IZDa ≥ 6 mm (yellow zone), additive when 6 mm > IZDa+p–IZDa ≥ 4 mm (green zone), indifferent when 4 mm > IZDa+p–IZDa > −6 mm (light pink zone) and negative when IZDa+p–IZDa ≤ − 6 mm (dark pink zone), where IZD corresponds to the inhibition zone diameter, a = antibiotic and p = phytochemical. Compound **2**, a nitrovinylfuran derivative, induced total growth inhibition against the RN4220 strain. More detailed information about the interactions is given in the Supplementary Materials, including some geometric shapes that might be overlayed on the graph (Supplementary Materials Table S1).

According to the results obtained through the disc diffusion method, the same combination of phytochemical-based molecules/antibiotics had different outcomes for each strain. For example, regarding the combination of the selected phytochemicals with fusidic acid (Figure 1), compound **2** had an additive effect against the strains MJMC102, MJMC109 and SA 1199B, an indifferent performance against strains Xu212 and RN4220, and a potentiation effect against strains CECT 976, MJMC110 and MJMC111. 

There were also differences in the outputs obtained for the same phytochemical combined with different antibiotics. For instance, there are some combinations where the same compound combined with a different antibiotic resulted in a different effect, varying from indifference to potentiation. For example, chalcone had an indifferent effect on fusidic acid against almost all strains; potentiation was found against MJMC110, with an additive effect against MJMC109 and MJMC111. On the other hand, chalcone potentiated or had an additive effect on mupirocin against most of the tested strains, with this combination being only considered indifferent against Xu212 and SA 1199B strains.

From Figure 1, it can be assumed that most of the molecules do not positively influence the effect of fusidic acid. Only chalcone, farnesol, compound **2**, and compound **3** had an effect on the action of fusidic acid. Chalcone potentiated the antibiotic activity against the MJMC110 strain. Farnesol potentiated the effect of fusidic acid against the two clinical isolates of MRSA (MJMC102 and MJMC111), and compound **3** potentiated the effect against the collection strain. The most promising compound with the most positive impact on the effect of fusidic acid was compound **2**, a nitrovinylfuran derivative. 

**Figure 2:**
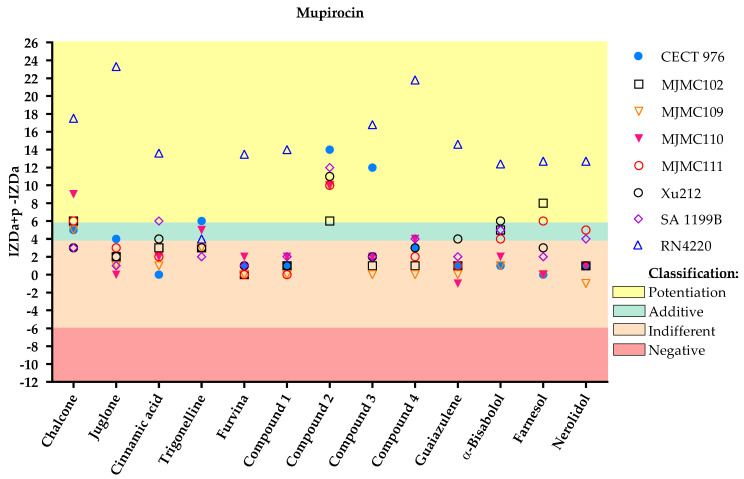
Inhibition zone diameter (IZD) values (mm) and respective classification according to the combined application of the selected phytochemicals and mupirocin against the eight different *S. aureus* strains. The effect of dual combinations of antibiotics and phytochemicals was classified as potentiation when IZDa+p–IZDa ≥ 6 mm (yellow zone), additive when 6 mm > IZDa+p –IZDa ≥ 4 mm (green zone), indifferent when 4 mm > IZDa+p–IZDa > −6 mm (light pink zone) and negative when IZDa+p–IZDa ≤ −6 mm (dark pink zone), where IZD corresponds to the inhibition zone diameter, a = antibiotic and p = phytochemical. Compound **2**, a nitrovinylfuran derivative, induced total growth inhibition against the RN4220 strain. More detailed information about the interactions is given in the Supplementary Materials, including some geometric shapes that might be overlayed on the graph (Table S1).

With respect to the effect of the 13 selected phytochemicals and derivatives on mupirocin (Figure 2), the behavior of compound **2** again stood out against all the bacterial strains. However, all the other molecules had at least one or two strains for which the combination had a beneficial antimicrobial effect. It is worth mentioning that almost all the molecules clearly potentiated the effect of mupirocin against the RN4220 strain. Only trigonelline had an additive interaction with mupirocin against that strain. Apart from compound **2**, only chalcone, α-bisabolol and nerolidol increased mupirocin activity or exhibited antimicrobial effects in combination with mupirocin against clinically isolated MRSA strains (MJMC102 and MJMC111).

**Figure 3:**
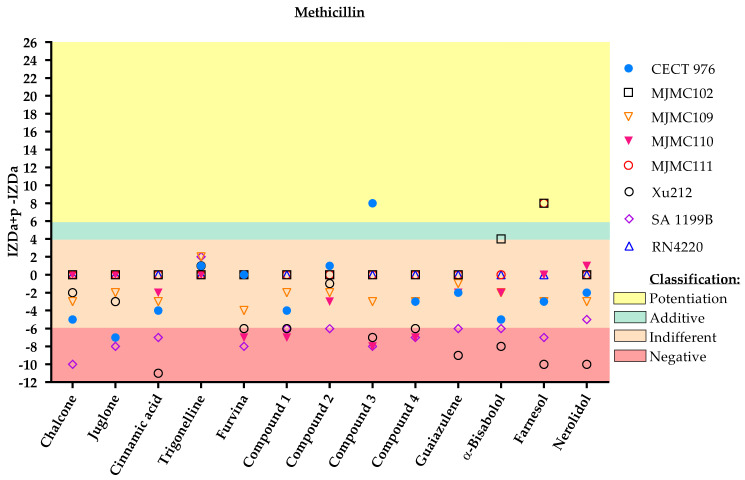
Inhibition zone diameter (IZD) values (mm) and respective classification according to the combined application of the selected phytochemicals and methicillin against the eight different *S. aureus* strains. The effect of dual combinations of antibiotics and phytochemicals was classified as potentiation when IZDa+p–IZDa ≥ 6 mm (yellow zone), additive when 6 mm > IZDa+p–IZDa ≥ 4 mm (green zone), indifferent when 4 mm > IZDa+p–IZDa > −6 mm (light pink zone) and negative when IZDa+p–IZDa ≤ −6 mm (dark pink zone), where IZD corresponds to the inhibition zone diameter, a = antibiotic and p = phytochemical. Compound **2**, a nitrovinylfuran derivative, induced total growth inhibition against the RN4220 strain. More detailed information about the interactions is given in the Supplementary Materials, including some geometric shapes that might be overlayed on the graph (Table S1).

**Figure 4:**
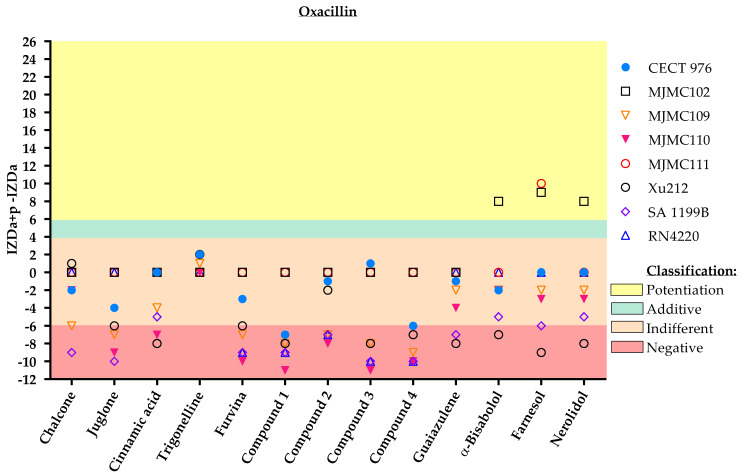
IZD values (mm) and respective classification according to the combined application of the selected phytochemicals and oxacillin against the eight different *S. aureus* strains. The effect of dual combinations of antibiotics and phytochemicals was classified as potentiation when IZDa+p–IZDa ≥ 6 mm (yellow zone), additive when 6 mm > IZDa+p–IZDa ≥ 4 mm (green zone), indifferent when 4 mm > IZDa+p–IZDa > −6 mm (light pink zone) and negative when IZDa+p–IZDa ≤ −6 mm (dark pink zone), where IZD corresponds to the inhibition zone diameter, a = antibiotic and p = phytochemical. Compound **2**, a nitrovinylfuran derivative, induced total growth inhibition against the RN4220 strain. More detailed information about the interactions is given in the Supplementary Materials, including some geometric shapes that might be overlayed on the graph (Table S1).

Although most of the molecules tested had modest effects when combined either with methicillin or oxacillin, some promising combinations were observed that may restore the effect of methicillin and/or oxacillin against MRSA strains (MJMC102 and MJMC111) (Figure 3 and Figure 4).

Farnesol was the molecule with the clearest potentiation effect on these antibiotics against the MRSA clinical isolates. The molecule α-bisabolol also potentiated or had an additive effect with methicillin and oxacillin against one of these strains (MJMC102), whereas nerolidol potentiated the effect of oxacillin against the MJMC102 strain.

**Figure 5:**
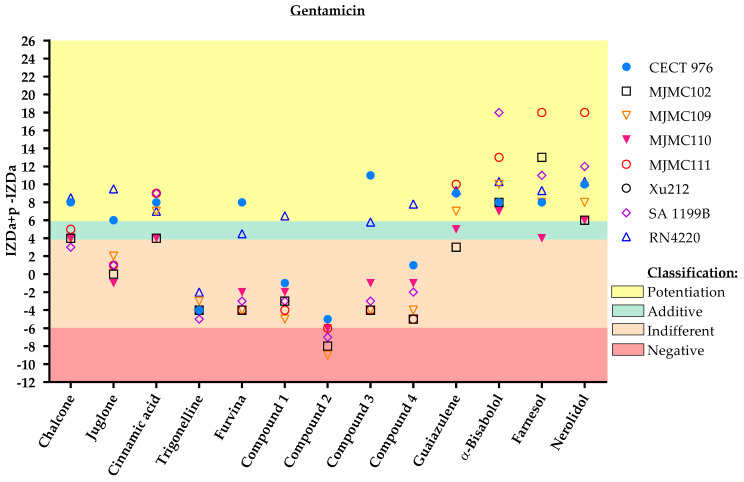
IZD values (mm) and respective classification according to the combined application of the selected phytochemicals and gentamicin against the eight different *S. aureus* strains. The effect of dual combinations of antibiotics and phytochemicals was classified as potentiation when IZDa+p –IZDa ≥ 6 mm (yellow zone), additive when 6 mm > IZDa+p–IZDa ≥ 4 mm (green zone), indifferent when 4 mm > IZDa+p–IZDa > −6 mm (light pink zone) and negative when IZDa+p–IZDa ≤ −6 mm (dark pink zone), where IZD corresponds to the inhibition zone diameter, a = antibiotic and p = phytochemical. Compound **2**, a nitrovinylfuran derivative, induced total growth inhibition against the RN4220 strain. More detailed information about the interactions is given in the Supplementary Materials, including some geometric shapes that might be overlayed on the graph (Table S1).

The results demonstrate that the sesquiterpenoid constituents of essential oils (guaiazulene, α-bisabolol, farnesol and nerolidol) potentiated the effect of gentamicin (Figure 5). In fact, only these phytochemicals and cinnamic acid were found to be possible potentiators of gentamicin against all the strains tested. Regarding the remaining molecules, they only potentiated or caused an additive effect against the collection-type strain (CECT 976) and RN4220.

## 3. Discussion

Plant secondary metabolites are organic compounds secreted to defend plants against herbivores and pathogen attack [12]. They are described to possess various physiological activities, including antioxidant, antidiabetic, antiproliferative, anti-inflammatory and antimicrobial activity [13]. Phytochemicals are described to exert their antimicrobial activity through mechanisms of action distinct from those of conventional antibiotics. Diverse studies have reported phytochemicals’ ability to inhibit cell wall synthesis, interfere with bacterial physiology through reduced membrane potential and lower levels of ATP synthesis, modulate antibiotic susceptibility by affecting bacterial resistance mechanisms and mitigate bacterial virulence through interference with bacterial communication and the establishment of complex microbial communities [12,13].

In this study, 13 phytochemicals, including chalcone, juglone, cinnamic acid, trigonelline, and four 2-nitrovinylfuran derivatives, guaiazulene, α-bisabolol, farnesol and nerolidol, were evaluated for their action on the modulation of bacterial antibiotic susceptibility. Combinations with five in-use antibiotics—fusidic acid, mupirocin, gentamicin, methicillin and oxacillin—were assessed through the disc diffusion method. The results demonstrated that among the antibiotics studied, there were some (i.e., mupirocin and gentamicin) whose effect was potentiated to a greater extent by the molecules selected. The other antibiotics (fusidic acid, methicillin and oxacillin) presented mostly an indifferent interaction. In general, oxacillin and methicillin were the antibiotics with the least potentiated effects overall. Still, it was on these antibiotics that occurred the most promising interactions, especially with the combination of some components of essential oils against MRSA strains (MJMC102 and MJMC111). Farnesol restored the activity of methicillin and oxacillin against both MRSA clinical isolates, whereas α-bisabolol and nerolidol potentiated the effect of oxacillin against MJMC102, an MRSA clinical strain. These results are in agreement with previous findings with respect to the importance of the combination of molecules to combat multidrug-resistant bacteria, in particular the combination of essential oils with existent antibiotics, aiming to bring them back to therapeutic efficacy [14,15,16]. Xi et al. described that some essential oils combined with specific antibiotics, particularly β-lactams, reduced antibiotic resistance in plasmid-conferred multidrug-resistant bacteria (*E. coli* J53 R1, *E. coli* J53 pMG309 and *E. coli* J53 pMG321) [17]. In that study, the authors found that peppermint, cinnamon bark and lavender essential oils could be used as antibiotic resistance-modifying agents [17]. El Atki et al. showed a synergistic effect between *Cinnamomum cassia* (cinnamon) essential oil and ampicillin or chloramphenicol against *S. aureus* (ATCC 25923) [18]. The authors stated that the combination of cinnamon essential oils and antibiotics can be used as a therapeutic application due to not only the synergistic effect observed but also a decrease in the minimum effective dose of the antibiotic [18]. In the present study, the results demonstrate that a possible synergy between phytochemicals and antibiotics is not only phytochemical-dependent but it is also antibiotic- and strain-dependent. For instance, if we analyze the results according to the antibiotic involved, it can be inferred that when looking to the potentiation of fusidic acid, the most positive interaction occurred with a nitrovinylfuran derivative (compound **2**) against almost all *S. aureus* strains used. This result is in line with our previous study, in which this compound was found to be the best *S. aureus* quorum-sensing inhibitor when compared with furvina and all the other nitrovinylfuran derivatives [19]. Its effect on quorum sensing prompted an increase in *S. aureus* susceptibility to fusidic acid [19]. 

By analyzing the phytochemical–antibiotic combination and by looking at the combination with mupirocin, the behavior of compound **2** stood out compared to that of the other molecules. This enhanced performance was followed by chalcone, α-bisabolol and farnesol but only against some specific strains. The results showed that all the molecules used, regardless of the class, potentiated the effect of mupirocin against the RN44220 strain. This strain contains a plasmid pUL5054, which carries the gene encoding the MsrA macrolide efflux protein, one of the efflux-related resistance mechanisms described for *S. aureus* [20]. When evaluating the combination of the molecules with methicillin and oxacillin, only farnesol was able to restore antibiotic action against the two MRSA clinical isolates. The molecule α-bisabolol also increased the effect of both antibiotics but only against one of the MRSA clinical isolates (MJMC102), whereas nerolidol only potentiated the effect of oxacillin against that same strain. Because all these positive interactions occur when components of essential oils are present, the results emphasize the potential of this class of phytochemicals for combinatorial therapy with antibiotics. In fact, this is supported by previous studies about the effects of essential oils and their constituents in the control of multidrug-resistant bacteria [21,22,23]. Kuroda et al. demonstrated that farnesol at a sub-MIC inhibited *S. aureus* lipase (SAL) against 8 methicillin-susceptible and 31 methicillin-resistant *S. aureus* clinical isolates [24]. SAL is an enzyme known to possess broad specificity towards triglycerides, which are molecules quite abundant on the human sebum [24]. The presence of this enzyme contributes to *S. aureus* skin colonization. Other studies indicated that farnesol inhibited fibrin fiber formation by inhibiting coagulase, which is also one of the most characteristic virulence factors of *S. aureus* [24,25,26]. Apart from that, nerolidol was also found to interfere with genes that regulate the pathogenicity of bacteria. It was reported that this molecule downregulated the expression of *hla*, an α-hemolysin gene related to a higher virulence of *S. aureus* [27]. Other studies have hypothesized that essential oils may alter antibiotic efflux pumps, which may end up restoring the effectiveness of some antibiotics that lose their clinical application [21]. For instance, α-bisabolol showed an ability to inhibit TetK and NorA efflux pumps in *S. aureus* strains [28]. These efflux pumps represent one of the mechanisms by which *S. aureus* exerts resistance against chemotherapeutic agents. Recently, a hypothesis was presented relating the increased virulence to the emergence of antibiotic resistance [29]. The correlation between virulence factors and the resistance to antimicrobials in *S. aureus* is being studied, and despite the complexity of this relationship, a synergistic interaction between these features seems to occur during infection [30].

Despite the huge variety of essential oils and their constituents, not all exhibit a strong antimicrobial effect, as differences in their chemical structure may influence antimicrobial response. Kon and Rai described that oxygenated terpenes exhibit higher antimicrobial activity than their hydrocarbon equivalents [21]. This statement is in accordance with our results, wherein the phytochemicals with the highest synergistic effects were farnesol, nerolidol and α-bisabolol. Guaiazulene demonstrated modest synergistic activity with most of the antibiotics tested. The differences obtained in the combined activity between the different constituents of essential oils and antibiotics may be explained by differences in their chemical structure. For instance, farnesol and nerolidol do not possess an aromatic ring, unlike guaiazulene and α-bisabolol. On the other hand, they contain hydroxyl groups in different numbers and positions and higher carbon chains. Because guaiazulene was the only phytochemical containing a fusion of cyclopentadiene and cycloheptatriene rings in its structure, the results obtained indicate that this particularity may decrease its antimicrobial activity. These phytochemicals, like other sesquiterpenoids, have high hydrophobicity, which facilitates penetration across the bacterial membrane and interaction with intracellular constituents [27]. However, even if there is a structural similarity among some of the molecules tested (i.e., nerolidol and farnesol), their antimicrobial action was different for the same strain, which demonstrated that strain variation may represent an important source of inconsistency in microbiological studies. In fact, when research findings refer to results of a certain microbiological strain, conclusions cannot be extended to other strains of the same species [31]. The results presented in this study demonstrate that the same molecule combined with the same antibiotic generated different outcomes depending on the strain involved, resulting in differences between synergy to antagonism for the same combination. These findings emphasize the importance of testing multiple strains under well-established conditions to enrich microbial risk assessment by providing new perspectives on strain variability. Despite that, the results reinforce the potential of essential oils for the development of synergistic combinations to increase the antibacterial effects of antibiotics against various strains of a multidrug-resistant bacterium.

Apart from the promise demonstrated in the use of essential oils and other classes of phytochemical-based molecules as antibiotic adjuvants, there is little knowledge on the molecular basis of these synergistic interactions to better understand their combined mechanism of action. Although the exact mechanism of action of the selected molecules, alone or in combination with antibiotics, was not evaluated in this study, their possible mechanism of action, especially of their chemical class, has already been reported in the literature [12,32,33]. It is believed that flavonoids inhibit ATPase activity and GrYB protein and elevate extracellular phosphatase and β-galactosidase, whereas chalcones were described to inhibit various efflux pumps (EPs) (e.g., EtBr EP and MexAB-OprM) [12]. The mode of action of essential oils varies according to the components of the extract. For instance, they were described to increase cell permeability; induce the leakage of cell constituents, alteration of the bacterial cell wall, membrane disturbance and ATP loss; inhibit protein synthesis; lead to pH disturbance, intracytoplasmic damage and DNA damage; and inhibit quorum sensing and biofilm formation [12,32]. Regarding phenolics, they were described to inhibit bacterial virulence factors, such as enzymes and toxins; interact with the cytoplasmic membrane; suppress biofilm formation; and exert a synergistic effect with antibiotics [33]. Despite this, much remains to be understood, as the mechanism of action of each component of the mixture is not necessarily the same for each alone, nor the sum of each one. The mechanism of action can be completely different when phytochemicals and antibiotics are used in combination.

The possible cytotoxic effect of the molecules alone or in combination is a research need. Some studies about possible toxic effects of the most promising molecules of this study have already been carried out by other authors. Among the most promising molecules, there were some whose toxicity was studied against specific cell lines and others that were once approved by FDA. Farnesol and nerolidol are examples of substances that were approved by FDA for distinct purposes, whereas the remaining were somehow already studied. Farnesol is reported in the literature as a safe substance and showed selective toxicity in damaged cells [34]. In vivo, farnesol exhibited a mean lethal dose (LD_50_) ≥ 5000 mg/kg when orally administered in rats or mice [34]. Nerolidol was also studied with respect to its toxicity against fibroblasts [35]. Mendanha et al. [35] concluded that this compound exhibited toxic effects towards this cell line at a concentration of 0.6 mM. α-bisabolol was also considered nontoxic to animals when orally administered in rats (LD_50_ = 14 g/kg), and it did not exhibit mutagenic effects [36]. Even if some cytotoxicity is found within the molecules in study and despite that in wound healing, there is no epidermal barrier, it is generally agreed that therapeutic concentrations are one or two orders of magnitude greater than in vitro cytotoxic concentrations, typically 4–12% *v/v* [37]. Apart from that, experiments about cytotoxicity should be performed only after choosing the best phytochemical–antibiotic combination because the cytotoxic effect of the combination is not necessarily the same as that of each of the substances alone.

When it comes to the conventional use of systemic antibiotics in the treatment of wound infections, there are some necessary changes that need to be put in place in clinical practice. There are studies supporting the effectiveness of topical antibiotics towards systemic ones against MRSA skin infections. For example, Lundberg and Frimodt-Møller evaluated topical and systemic application against MRSA skin infections on an experimental skin wound infection model in mice and compared the topical application of retapamulin (1%), fusidic acid (2%) and mupirocin (2%) with the systemic administration of linezolid (50–100 mg/kg/day) and vancomycin (50–200 mg/kg/day) twice daily for 3 days or 6 days [38]. Their findings suggested that topical treatment with retapamulin and mupirocin was significantly more effective than systemic treatment with linezolid and vancomycin in eradicating MRSA in skin wounds [38]. These studies clearly demonstrate that a shift in physicians’ mindsets is necessary, especially with a more routine inclusion of topical antibiotics instead of systemic ones for the treatment of skin wound infections. Although some concerns might appear around the topical application of antibiotics and resistance appearance, the inclusion of phytochemicals as resistance-modifying agents may overcome this challenge and bring back to life antibiotics that are no longer in use.

## 4. Conclusions

Antibiotic resistance is a serious public health threat that calls for a concerted global action. New treatment strategies to combat life-threatening infections, especially those caused by *S. aureus*, are urgently required. This problem, combined with the high probability of DFU development (up to 25%) in diabetic patients and the high incidence of infection development (more than half), raises the risk of an undesired result [5].

Our findings demonstrate that phytochemicals, a clearly underexploited resource, possess promising characteristics as antibiotic adjuvants and especially as antibiotic resistance-modifying agents. For instance, we demonstrated that farnesol, a sesquiterpenoid constituent of essential oils, at sub-MIC, was able to restore the activity of methicillin and oxacillin in two MRSA clinical isolates and potentiated the effect of the remaining antibiotics. Apart from that, we also emphasize the great potential of a more routine usage of topical antimicrobials in the treatment of DFU’s infections, regardless the strain in study.

## 5. Materials and Methods

### 5.1. Preparation of the Phytochemicals and Derivatives

Chalcone (Sigma-Aldrich, St. Louis, MO, USA), juglone (Sigma-Aldrich, St. Louis, MO, USA), cinnamic acid (Merck, Darmstadt, Germany), trigonelline (Sigma-Aldrich, St. Louis, MO, USA), guaiazulene (Sigma-Aldrich, St. Louis, MO, USA), α-bisabolol (Sigma-Aldrich, St. Louis, MO, USA), farnesol (Sigma-Aldrich, St. Louis, MO, USA) and nerolidol (Sigma-Aldrich, St. Louis, MO, USA) were purchased as pure compounds. Furvina and 2-nitrovinylfuran derivatives 1–4 were synthesized as previously described [19,39]. Stock solutions of trigonelline were prepared in sterile distilled water, whereas for the remaining molecules, dimethyl sulfoxide (DMSO, 100%) was used as the solvent. For phytochemical-based molecules, serial dilutions from 1000 mg/L to 6.25 mg/L were prepared when needed. The percentage of DMSO never exceeded 10% (*v/v*) of the final volume. 

### 5.2. Preparation of Antibiotics

Fusidic acid (Sigma-Aldrich, St. Louis, MO, USA), mupirocin (AppliChem, GmbH, Darmstadt, Germany), methicillin (Thermo Fisher Scientific, Waltham, MA, USA), oxacillin (Sigma-Aldrich, St. Louis, MO, USA) and gentamicin (AppliChem, GmbH, Darmstadt, Germany) were also purchased as pure compounds. Stock solutions of fusidic acid, methicillin, oxacillin and gentamicin were prepared in sterile distilled water. For mupirocin, the stock solution was prepared in dimethyl sulfoxide (DMSO, 100%). The percentage of DMSO never exceeded 10% (*v/v*) of the final volume. The mass of antibiotics on the disc used was selected according to Clinical and Laboratory Standards Institute (CLSI) guidelines (fusidic acid: 10 µg/disc; mupirocin: 200 µg/disc; methicillin: 5 µg/disc; oxacillin: 1 µg/disc; gentamicin: 10 µg/disc).

### 5.3. Bacterial Strains

Eight different *S. aureus* strains were selected for this study: CECT 976 (Spanish Type Culture Collection strain); tetracycline-resistant strain Xu212 (methicillin resistant *S. aureus* strain and TetK tetracycline efflux protein); SA 1199B, which is a strain that overexpresses the NorA gene encoding the NorA multidrug-resistant efflux pump; RN4220, which expresses MsrA macrolide efflux protein; and four clinical isolates from foot wounds, i.e., MJMC102, MJMC109, MJMC110 and MJMC111. MJMC102 and MJMC111 are two methicillin-resistant *S. aureus* strains, whereas MJMC109 and MJMC110 are methicillin-susceptible. The clinical isolates belong to the MJMC collection and were isolated from diabetic foot ulcer exudates of patients hospitalized in diverse departments of Hospital Centre of Trás-os-Montes and Alto Douro (CHTMAD), located in the north of Portugal. The study was granted approval by the Ethics Committee of CHTMAD according to a protocol established in 2004.

### 5.4. Determination of Minimum Inhibitory Concentration (MIC) and Minimum Bactericidal Concentration (MBC)

The antimicrobial effect of all phytochemicals against *S. aureus* collection type (CECT 976) and clinical isolates (including overexpressed efflux pumps and MSSA/MRSA) was evaluated. For this study, the *S. aureus* strains were inoculated aerobically overnight in Mueller–Hinton broth (MHB; Merck, Darmstadt, Germany) at 37 °C at 150 rpm.

The MIC and MBC of all the phytochemicals were determined based on the microdilution method as previously described [40]. This method comprises an overnight grown bacteria in medium and then an adjustment of the optical density (OD) to 0.132 ± 0.02 (λ = 600 nm). Then, a volume of 180 μL of this cell suspension was added to sterile, flat, clear-bottomed polystyrene (PS) 96-well microtiter plates (Orange Scientific, Braine-l’Alleud, Belgium), already containing 20 μL of the compound at concentrations from 1000 to 6.25 mg/L. In the end, the volume of each compound never exceeded 10% (*v/v*) of the well volume. Microtiter plates were then incubated for 24 h at 37 °C under agitation (150 rpm). Absorbance measurements were performed at the beginning (t = 0 h) and at the end (t = 24 h) of the incubation period using a microplate reader (Synergy HT, Biotek, Winooski, VT, USA). Cell suspensions with and without DMSO were used as controls to assess the effect of DMSO on cell growth. MIC was set as the lowest concentration of the compound at which the final OD was equal to or lower than the initial OD (cell growth inhibition). This test was performed three times with three replicates.

To assess the MBC, a volume of 10 μL of the well content corresponding to the phytochemical’s concentration equal to and above the MIC was plated in agar plates and left to incubate for 24 h at 37 °C. The MBC of each compound was the lowest concentration tested at which a total inhibition of bacterial growth was observed [40]. This experiment was performed at least in triplicate with three replicates.

### 5.5. Antibiotic/Phytochemical Dual Combination: Disc Diffusion Method

The study of the antimicrobial effects of phytochemicals combined with antibiotics was performed by a modification of the disc diffusion assay, according to Abreu et al. [41]. In this method, the natural compounds were added to MHB agar (at 0.1 × MIC or at 1000 mg/L in cases where MIC was not detected) after autoclavation and a medium cooling step (medium temperature, approximately 40 °C) to avoid its deterioration. Portions of bacterial colonies were picked from overnight cultures in MH solid medium. The suspension of bacteria was prepared with 0.85% NaCl, which was adjusted to meet 0.5 McFarland turbidity standards (OD_600_ = 0.132). The suspension was spread with a sterile cotton swap into a Petri dish (90 mm in diameter) containing 20 mL of Mueller–Hinton agar (MHA), with each phytochemical incorporated at a specified concentration. MHA plates without incorporated phytochemicals were used as controls. Sterile filter paper discs (6 mm in diameter) impregnated with 15 µL of antibiotics were placed on the agar plate seeded with the respective bacteria. Discs impregnated with DMSO were used as negative controls because most of the phytochemicals and mupirocin were dissolved in this reagent. After 24 h of incubation at 37 °C, the diameter of the inhibition halo (clear zones without bacterial growth) was measured. No inhibition halo was found on the discs impregnated with DMSO. All tests were performed in triplicate, and the antibacterial activity was expressed as the mean of inhibition zone diameters (IZD, mm).

#### Classification

The effect of dual combinations of antibiotics and phytochemicals can be classified according to Abreu et al. [41]:

▪ Potentiation (+++): (IZDa+p–IZDa) ≥ 6 mm;
▪ Additive (++): 6 mm > (IZDa+p–IZDa) ≥ 4 mm;
▪ Indifferent (+): 4 mm > (IZDa+p–IZDa) > −6 mm;
▪ Negative (–): (IZDa+p–IZDa) ≤ −6 mm,

where IZD corresponds to the inhibition zone diameter, a = antibiotic and p = phytochemicals.

## Figures and Tables

**Table 1:** Selected phytochemicals and derivatives, as well as antibiotics, with their respective class and chemical structure.

	Class	Compound	Chemical Structure
Phytochemicals	Phenolics	Chalcone	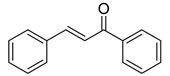
		Juglone	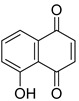
		Cinnamic acid	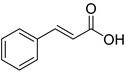
	Alkaloid	Trigonelline	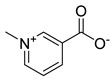
	Synthetic nitrovinylfuran	Furvina	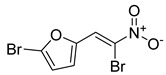
	2-nitrovinylfuran derivatives	Compound **1**	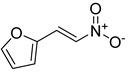
		Compound **2**	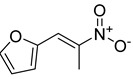
		Compound **3**	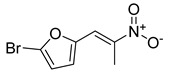
		Compound **4**	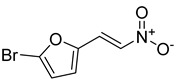
	Sesquiterpenoid constituents of essential oils	Guaiazulene	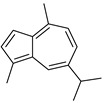
		α-bisabolol	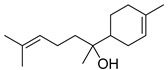
		Farnesol	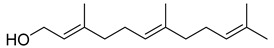
		Nerolidol	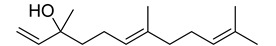
Antibiotics	Fusidane	Fusidic acid	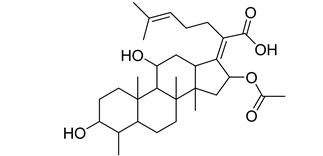
	Carboxylic acid	Mupirocin	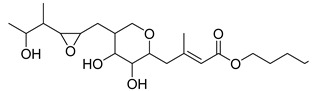
	Penicillin	Methicillin	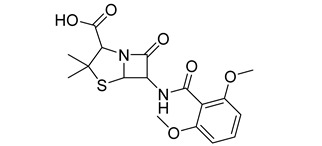
		Oxacillin	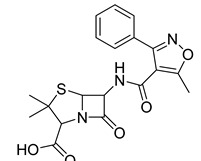
	Aminoglycoside	Gentamicin	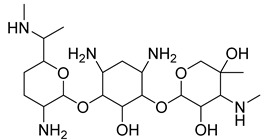

**Table 2:** MIC and MBC (mg/mL) values of the selected phytochemical-based molecules for *S. aureus* collection (CECT 976), *S. aureus* clinical isolates (MJMC102, MJMC109, MJMC110 and MJMC111) and *S. aureus* strains with known resistance patterns (Xu212, SA 1199B and RN4220).

		CECT 976		MJMC102		MJMC109		MJMC110		MJMC111		Xu212		SA 1199B		RN4220	
		MIC	MBC	MIC	MBC	MIC	MBC	MIC	MBC	MIC	MBC	MIC	MBC	MIC	MBC	MIC	MBC
Chalcone		100	>1000	200	>1000	200	>1000	200	>1000	200	>1000	200	>1000	200	>1000	200	>1000
Juglone		12.5	50	12.5–25	50	12.5–25	25	12.5	25	12.5	50	12.5	50	12.5–25	50	25	50
Cinnamic acid		>1000	>1000	>1000	>1000	>1000	>1000	>1000	>1000	>1000	>1000	>1000	>1000	>1000	>1000	>1000	>1000
Trigonelline		>1000	>1000	>1000	>1000	>1000	>1000	>1000	>1000	>1000	>1000	>1000	>1000	>1000	>1000	>1000	>1000
Furvina		100	200	25	200	25	100	25	200	25	200	25	100	25	50	25	50
2-nitrovinylfuran derivatives	Compound **1**	50	400	100	1000	200	800	200	>1000	200	800	200	800	200	800	50	400
	Compound **2**	>1000	>1000	>1000	>1000	>1000	>1000	>1000	>1000	>1000	>1000	>1000	>1000	>1000	>1000	>1000	>1000
	Compound **3**	400	800	100	>1000	100	>1000	100	>1000	100	>1000	100	>1000	100	>1000	100	>1000
	Compound **4**	>1000	>1000	200	1000	200	800	200	>1000	200	800	200	800	200	800	50	400
Guaiazulene		200	>1000	200	>1000	200	>1000	200	>1000	200	>1000	100	>1000	100	>1000	200	>1000
α-Bisabolol		50	>1000	100	>1000	50	>1000	100	>1000	100	>1000	100	>1000	100	>1000	100	>1000
Farnesol		25	50	100	800	25	800	25	800	50	800	25	25	25	25	100	>1000
Nerolidol		100	>1000	100	>1000	50	>1000	100	>1000	100	>1000	100	>1000	100	>1000	100	>1000

## Data Availability

Not applicable.

## Supplementary Materials

The following are available online at https://www.mdpi.com/article/10.3390/antibiotics11050620/s1, Table S1: Inhibition zone diameters and respective classification of the results for the combinatorial application of different tested phytochemicals and antibiotics.
