# Supplementary material for: Screening of Natural Molecules as Adjuvants to Topical Antibiotics to Treat Staphylococcus aureus from Diabetic Foot Ulcer Infections
Source: Antibiotics (Basel). 2022 May 4;11(5):620. doi: 10.3390/antibiotics11050620 (PMC9137705; doi:10.3390/antibiotics11050620)
Supplement: Supplementary file 1 [file antibiotics-11-00620-s001.zip › antibiotics-1693356-supplementary.pdf]

Table S1. Inhibition zone diameters and respective classification of the results for the combinatorial application of different phytochemicals and antibiotics. Classification (C): **Potentiation (+++):** (IZDa+p –IZDa) ≥ 6 mm; **Additive (++):** 6 mm > (IZDa+p –IZDa) ≥ 4 mm; **Indifferent (+):** 4 mm > (IZDa+p –IZDa) > -6 mm; **Negative (-):** (IZDa+p –IZDa) ≤ - 6 mm. **T.I.** – total inhibition.

Antibiotic	Phytochemical	Antibiotic mass (µg/disc)/ Phytochemical concentration (µg/mL)	CECT 976			SA102			SA109			SA110			SA111			Xu212			1199B			RN4220		
			IZD_a (mm)	IZD_{a+p} (mm)	C	IZD_a (mm)	IZD_{a+p} (mm)	C	IZD_a (mm)	IZD_{a+p} (mm)	C	IZD_a (mm)	IZD_{a+p} (mm)	C	IZD_a (mm)	IZD_{a+p} (mm)	C	IZD_a (mm)	IZD_{a+p} (mm)	C	IZD_a (mm)	IZD_{a+p} (mm)	C	IZD_a (mm)	IZD_{a+p} (mm)	C
Fusidic acid	Chalcone	10/10	25.0	26.0	+											x										
		10/20		x		24.0	27.0	+	22.0	25.5	++	**25.0**	**32.5**	**+++**	24.0	27.8	++	0	0	+	24.0	26.3	+	0	0	+
	Juglone	10/1.25	25.0	25.3	+	24.0	26.0	+	22.0	25.0	+	25.0	24.0	+	24.0	24.5	+	0	0	+	24.0	26.0	+		x	
		10/2.5											x											0	0	+
	Cinnamic acid	10/100	27.0	28.8	+	24.3	26.2	+	24.2	26.2	+	25.3	27.3	+	25.7	28.0	+	0	0	+	24.0	26.7	+	0	0	+
	Trigonelline	10/1000	25.0	30.0	++	24.0	27.0	+	22.0	27.0	++	25.0	27.8	+	24.0	26.3	+	0	0	+	24.0	26.5	+	0	0	+
	Furvina	10/2.5		x		26.3	25.5	+	24.5	23.8	+	24.8	25.3	+	26.0	27.0	+	0	0	+	25.8	23.5	+	0	0	+
		10/10	28.8	28.0	+											x										
	Compound 1	10/5	28.8	28.0	+									x										0	0	+
		10/10				26.3	25.0	+									x									
		10/20							24.5	24.8	+	24.8	24.8	+	26.0	27.3	+	0	0	+	25.8	25.5	+		x	
	Compound 2	10/1000	**28.8**	**35.3**	**+++**	26.3	30.0	++	24.5	29.5	++	**24.8**	**31.3**	**+++**	**26.0**	**34.0**	**+++**	0	0	+	25.8	29.5	++	**0**	**T.I.**	**+++**
	Compound 3	10/10				26.3	26.4	+	24.5	23.8	+	24.8	25.0	+	26.0	26.8	+	0	0	+	25.8	24.8	+		x	
		10/40	**28.8**	**39.3**	**+++**											x										
	Compound 4	10/5											x											0	0	+
		10/20				26.3	25.3	+	24.5	21.8	+	24.8	24.8	+	26.0	26.5	+	0	0	+	25.8	26.8	+		x	
		10/100	28.8	30.0	+											x										
	Guaiazulene	10/10								x								0	0	+	24.0	24.5	+		x	
		10/20	27.0	25.5	+	24.3	26.3	+	24.2	24.7	+	25.3	23.3	+	25.7	27.8	+			x				0	0	+
	α-Bisabolol	10/5	27.0	25.8	+		x		24.2	28.8	++								x							
		10/10		x		24.3	26.8	+		x		25.3	26.0	+	25.7	28.7	+	0	0	+	24.0	28.3	++	0	0	+

	Farnesol	10/2.5	27.0	25.5	+		x		24.2	25.8	+	25.3	25.5	+		x		0	0	+	24.0	26.5	+		x	
		10/5						x							**25.7**	**31.3**	**+++**					x				
		10/10		x		**24.3**	**32.3**	**+++**								x								0	0	+
	Nerolidol	10/5			x				24.2	22.0	+								x							
		10/10	27.0	27.0	+	24.3	22.3	+		x		25.3	24.0	+	25.7	25.8	+	0	0	+	24.0	25.8	+	0	0	+
Mupirocin	Chalcone	200/10	35.8	40.5	++											x										
		200/20		x		**33.0**	**39.3**	**+++**	35.5	40.3	++	**34.5**	**43.8**	**+++**	**31.3**	**36.8**	**+++**	37.0	40.3	+	38.0	41.0	+	**6.0**	**23.5**	**+++**
	Juglone	200/1.25	35.8	39.3	++	33.0	34.5	+	35.5	36.8	+	34.5	35.0	+	31.3	34.0	+	37.0	38.5	+	38.0	38.5	+	**6.0**	**29.5**	**+++**
		200/2.5											x											6.0	10.0	++
	Cinnamic acid	200/100	39.5	39.0	+	32.1	35.3	+	35.8	36.7	+	36.1	38.0	+	33.2	34.8	+	36.8	41.3	++	**36.1**	**41.8**	**+++**	**4.8**	**18.3**	**+++**
	Trigonelline	200/1000	**35.8**	**41.8**	**+++**	33.0	35.8	+	35.5	38.8	+	34.5	39.3	++	31.3	34.0	+	37.0	39.8	+	38.0	40.0	+		x	
	Furvina	200/2.5				33.3	33.0	+	34.8	34.8	+	35.0	37.0	+	33.5	33.8	+	37.3	38.3	+	35.5	36.5	+	**5.8**	**19.3**	**+++**
		200/10	40.3	41.8	+											x										
	Compound 1	200/5	40.3	41.0	+									x										**5.8**	**19.8**	**+++**
		200/10		x		33.3	34.5	+									x									
		200/20			x				34.8	35.0	+	35.0	37.3	+	33.5	33.5	+	37.3	37.8	+	35.5	37.3	+		x	
	Compound 2	200/1000	**40.3**	**54.0**	**+++**	**33.3**	**39.5**	**+++**	**34.8**	**44.3**	**+++**	**35.0**	**45.0**	**+++**	**33.5**	**43.8**	**+++**	**37.3**	**47.8**	**+++**	**35.5**	**47.8**	**+++**	**5.8**	**T.I.**	**+++**
	Compound 3	200/10				33.3	34.5	+	34.8	34.8	+	35.0	37.3	+	33.5	35.5	+	37.3	39.5	+	35.5	37.5	+	**5.8**	**22.5**	**+++**
		200/40	**40.3**	**52.0**	**+++**											x										
	Compound 4	200/5											x											**5.8**	**27.5**	**+++**
		200/20		x		33.3	34.0	+	34.8	34.8	+	35.0	38.8	++	33.5	35.0	+	37.3	40.0	+	35.5	39.5	++		x	
		200/100	40.3	43.3	+											x										
	Guaiazulene	200/10								x								36.8	40.3	++	36.1	38.3	+		x	
		200/20	39.5	40.5	+	32.1	33.3	+	35.8	35.7	+	36.1	35.0	+	33.2	33.7	+			x				**4.8**	**19.4**	**+++**
	α-Bisabolol	200/5	39.5	41.0	+		x		35.8	36.5	+								x							
		200/10		x		32.1	36.8	++		x		36.1	37.8	+	33.2	36.7	++	**36.8**	**42.7**	**+++**	36.1	40.7	++	**4.8**	**17.2**	**+++**
	Farnesol	200/2.5	39.5	39.8	+		x		35.8	36.0	+	36.1	36.3	+		x		36.8	40.3	+	36.1	38.0	+		x	
		200/5						x							**33.2**	**38.9**	**+++**					x				
		200/10		x		**32.1**	**40.2**	**+++**								x								**4.8**	**17.5**	**+++**
	Nerolidol	200/5			x				35.8	35.0	+								x							
		200/10	39.5	40.5	+	32.1	33.0	+		x		36.1	37.5	+	33.2	38.0	++	36.8	37.5	+	36.1	39.8	++	**4.8**	**17.5**	**+++**

	Chalcone	5/10	14.8	10.3	+											x										
		5/20		x		0	0	+	9.0	6.5	+	5.5	7.0	+	0	0	+	6.0	4.3	+	13.0	3.0	-	0	0	+
	Juglone	5/1.25	14.8	8.0	-	0	0	+	9.0	6.8	+	5.5	0	-	0	0	+	6.0	3.5	+	13.0	5.0	-		x	
		5/2.5											x											0	0	+
	Cinnamic acid	5/100	20.5	17.0	+	0	0	+	14.3	12.0	+	12.8	10.3	+	0	0	+	21.5	10.5	-	19.0	12.3	-	0	0	+
	Trigonelline	5/1000	14.8	15.3	+	0	0	+	9.0	10.5	+	5.5	8.5	+	0	0	+	6.0	7.0	+	13.0	15.0	+	0	0	+
	Furvina	5/2.5				0	0	+	13.5	10.0	+	14.5	7.5	-	0	0	+	11.5	8.5	-	18.0	10.0	-	0	0	+
		5/10	18.2	18.3	+											x										
		5/5	18.2	14.3	+									x										0	0	+
	Compound 1	5/10				0	0	+									x									
		5/20							13.5	11.3	+	14.5	7.5	-	0	0	+	11.5	8.3	-	18.0	12.0	-		x	
	Compound 2	5/1000	18.2	19.0	+	0	0	+	13.5	11.3	+	14.5	11.3	+	0	0	+	11.5	12.8	+	18.0	12.0	-	**0**	**T.I.**	**+++**
Methicillin	Compound 3	5/10				0	0	+	13.5	10.8	+	14.5	7.0	-	0	0	+	11.5	7.3	-	18.0	9.8	-	0	0	+
		5/40	**18.2**	**26.0**	**+++**											x										
		5/5											x											0	0	+
	Compound 4	5/20		x		0	0	+	13.5	11.0	+	14.5	7.8	-	0	0	+	11.5	8.0	-	18.0	11.0	-		x	
		5/100	18.2	15.0	+											x										
	Guaiazulene	5/10								x								21.5	12.3	-	19.0	13.0	-		x	
		5/20	20.5	18.5	+	0	0	+	14.3	11.8	+	12.8	11.0	+	0	0	+			x				0	0	+
	α-Bisabolol	5/5	20.5	16.0	+		x		14.3	11.0	+								x							
		5/10		x		0	3.5	++		x		12.8	11.0	+	0	0	+	21.5	13.5	-	19.0	12.8	-	0	0	+
	Farnesol	5/2.5	20.5	18.0	+		x		14.3	11.5	+	12.8	13.0	+		x		21.5	11.8	-	19.0	12.5	-		x	
		5/5						x							**0**	**7.8**	**+++**					x				
		5/10		x		**0**	**8.0**	**+++**								x								0	0	+
	Nerolidol	5/5			x				14.3	13.3	+								x							
		5/10	20.5	18.8	+	0	0	+		x		12.8	13.5	+	0	0	+	21.5	12.0	-	19.0	12.8	+	0	0	+
Oxacillin	Chalcone	1/10	20.0	18.0	+											x										
		1/20		x		0	0	+	17.0	11.3	-	15.5	13.3	+	0	0	+	12.5	13.0	+	20.8	12.0	-	0	0	+
	Juglone	1/1.25	20.0	16.0	+	0	0	+	17.0	10.0	-	15.5	6.8	-	0	0	+	12.5	7.0	-	20.8	11.3	-	0	0	+

		1/2.5											x											0	0	+
	Cinnamic acid	1/100	20.8	20.3	+	0	0	+	15.5	11.8	+	15.8	9.3	-	0	0	+	21.8	14.0	-	19.5	15.0	+	0	0	+
	Trigonelline	1/1000	20.0	21.8	+	0	0	+	17.0	17.5	+	15.5	15.3	+	0	0	+	12.5	14.5	+	20.8	22.3	+		x	
	Furvina	1/2.5		x		0	0	+	18.3	11.8	-	17.8	7.8	-	0	0	+	14.0	7.8	-	21.3	12.3	-	0	0	+
		1/10	22.5	20.0	+											x										
	Compound 1	1/5	22.5	15.3	-									x										0	0	+
		1/10				0	0	+									x									
		1/20							18.3	10.8	-	17.8	6.8	-	0	0	+	14.0	6.3	-	21.3	12.0	-		x	
	Compound 2	1/1000	22.5	21.2	+	0	0	+	18.3	11.3	-	17.8	10.3	-	0	0	+	14.0	11.8	+	21.3	14.0	-	0	**T.I.**	**+++**
	Compound 3	1/10				0	0	+	18.3	10.3	-	17.8	7.0	-	0	0	+	14.0	5.8	-	21.3	11.3	-	0	0	+
		1/40	22.5	23.3	+											x										
	Compound 4	1/5											x											0	0	+
		1/20				0	0	+	18.3	9.5	-	17.8	7.8	-	0	0	+	14.0	7.3	-	21.3	11.8	-		x	
		1/100	22.5	16.5	-											x										
	Guaiazulene	1/10								x								21.8	13.5	-	19.5	13.0	-		x	
		1/20	20.8	20.3	+	0	0	+	15.5	13.3	+	15.8	12.0	+	0	0	+			x				0	0	+
	α-Bisabolol	1/5	20.8	18.8	+				15.5	13.3	+								x							
		1/10		x		**0**	**8.0**	**+++**		x		15.8	14.3	+	0	0	+	21.8	14.8	-	19.5	14.5	+	0	0	+
	Farnesol	1/2.5	20.8	20.8	+				15.5	13.5	+	15.8	12.5	+		x		21.8	13.0	-	19.5	14.0	-		x	
		1/5						x							**0**	**10.3**	**+++**					x				
		1/10		x		**0**	**9.3**	**+++**								x								0	0	+
	Nerolidol	1/5			x				15.5	13.3	+								x							
		1/10	20.8	21.0	+	**0**	**8.3**	**+++**		x		15.8	13.0	+	0	0	+	21.8	14.0	-	19.5	14.8	+	0	0	+
	Chalcone	10/10	**12.0**	**19.5**	**+++**											x										
		10/20		x		13.3	16.8	++	11.0	15.0	++	12.5	16.8	++	13.3	18.5	++	0	0	+	13.3	16.0	+	**8.8**	**17.3**	**+++**
	Juglone	10/1.25	**12.0**	**17.8**	**+++**	13.3	13.3	+	11.0	12.8	+	12.5	12.0	+	13.3	14.0	+	0	0	+	13.3	14.0	+			
		10/2.5											x											**8.8**	**18.3**	**+++**
Gentamicin	Cinnamic acid	10/100	**18.3**	**26.0**	**+++**	17.5	21.3	++	**13.0**	**19.8**	**+++**	13.8	17.5	++	**15.2**	**24.0**	**+++**	0	0	+	**16.3**	**24.8**	**+++**	**15.8**	**22.8**	**+++**
	Trigonelline	10/1000	12.0	8.0	+	13.3	9.8	+	11.0	7.8	+	12.5	8.8	+	13.3	9.8	+	0	0	+	13.3	8.0	+	8.8	7.3	+
	Furvina	10/2.5		x		17.3	13.8	+	15.5	11.8	+	14.3	12.0	+	18.3	14.0	+	0	0	+	16.3	13.0	+	13.8	18.3	++
		10/10	**18.3**	**26.0**	**+++**											x										

	10/5	18.3	17.8	+									x										**13.8**	**20.3**	**+++**
Compound 1	10/10		x		17.3	14.3	+									x									
	10/20			x				15.5	10.8	+	14.3	12.5	+	18.3	14.0	+	0	0	+	16.3	13.0	+		x	
Compound 2	10/1000	18.3	13.4	+	17.3	9.5	-	15.5	6.5	-	14.3	8.0	-	18.3	12.0	-	0	0	+	16.3	9.8	-	**13.8**	**T.I.**	**+++**
Compound 3	10/10		x		17.3	13.8	+	15.5	11.5	+	14.3	13.3	+	18.3	14.3	+	0	0	+	16.3	13.5	+	**13.8**	**19.5**	**+++**
	10/40	**18.3**	**29.0**	**+++**											x										
	10/5											x											**13.8**	**21.5**	**+++**
Compound 4	10/20				17.3	12.3	+	15.5	12.0	+	14.3	13.5	+	18.3	13.8	+	0	0	+	16.3	14.8	+		x	
	10/100	18.3	19.8	+											x										
Guaiazulene	10/10								x								0	0	+	**16.3**	**25.0**	**+++**		x	
	10/20	**18.3**	**27.0**	**+++**	17.5	20.5	+	**13.0**	**19.5**	**+++**	13.8	18.5	++	**15.2**	**25.0**	**+++**			x				**15.8**	**25.0**	**+++**
α-Bisabolol	10/5	**18.3**	**25.8**	**+++**		x		**13.0**	**23.0**	**+++**								x							
	10/10		x		**17.5**	**25.0**	**+++**		x		**13.8**	**21.0**	**+++**	**15.2**	**28.5**	**+++**	0	0	+	**16.3**	**34.0**	**+++**	**15.8**	**26.0**	**+++**
	10/2.5	**18.3**	**26.0**	**+++**		x		**13.0**	**20.5**	**+++**	13.8	18.0	++		x		0	0	+	**16.3**	**27.0**	**+++**		x	
Farnesol	10/5						x							**15.2**	**33.0**	**+++**					x				
	10/10		x		**17.5**	**30.0**	**+++**								x								**15.8**	**25.0**	**+++**
Nerolidol	10/5			x				**13.0**	**21.3**	**+++**								x							
	10/10	**18.3**	**28.5**	**+++**	**17.5**	**23.3**	**+++**		x		**13.8**	**19.5**	**+++**	**15.2**	**32.8**	**+++**	0	0	+	**16.3**	**28.5**	**+++**	**15.8**	**26.0**	**+++**
